# Tubo-ovarian abscess with sepsis in a nonagenarian woman: a case report and literature review

**DOI:** 10.1186/s12905-019-0782-6

**Published:** 2019-06-19

**Authors:** Kuan-Yi Chen, Jen-Yu Tseng, Chih-Yu Yang

**Affiliations:** 1 Department of Chest Medicine, Taipei Veterans General Hospital, Taipei, Taiwan; 2 Department of Obstetrics and Gynecology, Taipei Veterans General Hospital, Taipei, Taiwan; 3 Division of Nephrology, Department of Medicine, Taipei Veterans General Hospital, Taipei, Taiwan; 4 Institute of Clinical Medicine, School of Medicine, National Yang-Ming University, Taipei, Taiwan; 5 Stem Cell Research Center, National Yang-Ming University, Taipei, Taiwan; 6 School of Medicine, National Yang-Ming University, No. 201, Section 2, Shih-Pai Road, Taipei, 11217 Taiwan

**Keywords:** Fever of unknown origin, Tubo-ovarian abscess, Post-menopausal, Nonagenarian, Chronic kidney disease, Case report

## Abstract

**Background:**

A complete infectious focus survey relies on a thorough physical examination as well as a pelvic examination. Tubo-ovarian abscess, though less likely to occur in senior women, may become a life-threatening disease requiring emergent surgery. Hence, clinical awareness and aggressive management are warranted to avoid delayed diagnosis and subsequent complications.

**Case presentation:**

We report a post-menopausal woman presented with sepsis of unknown origin, which turned out to be a huge tubo-ovarian abscess. Although tubo-ovarian abscess mostly occurs in women of fertile age, it is likely that the immune status of our post-menopausal patient was compromised because of old age and uremia. Moreover, due to underlying dementia, she could not express her discomfort in the early stage. Her sepsis resolved after a unilateral salpingo-oophorectomy surgery and antibiotic treatment. It is crucial to exclude pelvic inflammatory disease (PID) if no specific source of infection can be identified.

**Conclusions:**

Rupture of the tubo-ovarian abscess is a condition of high mortality rate. Although tubo-ovarian abscess is more likely to develop in patients aged 15–25 years old, the tubo-ovarian abscess should be listed as a differential diagnosis in all post-menopausal women, especially those who are immunocompromised or with a palpable pelvic mass, to enable timely management and better prognosis.

## Background

Tubo-ovarian abscess, one entity of pelvic inflammatory diseases (PID), mostly occurs in women of fertile age and may become a life-threatening condition requiring emergent surgery. Therefore, in order to ensure early recognition, it is essential to exclude PID if no specific source of infection can be identified, even in elderly post-menopausal women. In this report, we highlight the pivotal role of pelvic examination in a thorough infectious focus work-up.

## Case presentation

A 91-year-old post-menopausal woman without diabetes mellitus or hypertension presented with shortness of breath, fever up to 38.5 degrees, anuria, and conscious disturbance for two days. Tracing back her history, she has dementia for 20 years with chronic kidney disease in stage 5, and she has not received any bowel or adnexal surgery. Last year, a transvaginal ultrasound had been performed by the gynecologist for a palpable pelvic mass, but only endometrial hyperplasia was impressed. Upon this admission, physical examination revealed a palpable mass as well, but there was no evident tenderness initially. Her body mass index was 23 Kg/m^2^. Laboratory test showed leukocytosis, azotemia with blood urea nitrogen 117 mg/dL, creatinine 12.9 mg/dL, C-reactive protein 26.2 mg/dL, procalcitonin 2.5 ng/mL, and pyuria. We initiated hemodialysis therapy for her uremia. Stool routine and culture showed negative results, indicating that colitis or gastrointestinal bleeding is less likely.

**Fig. 1:**
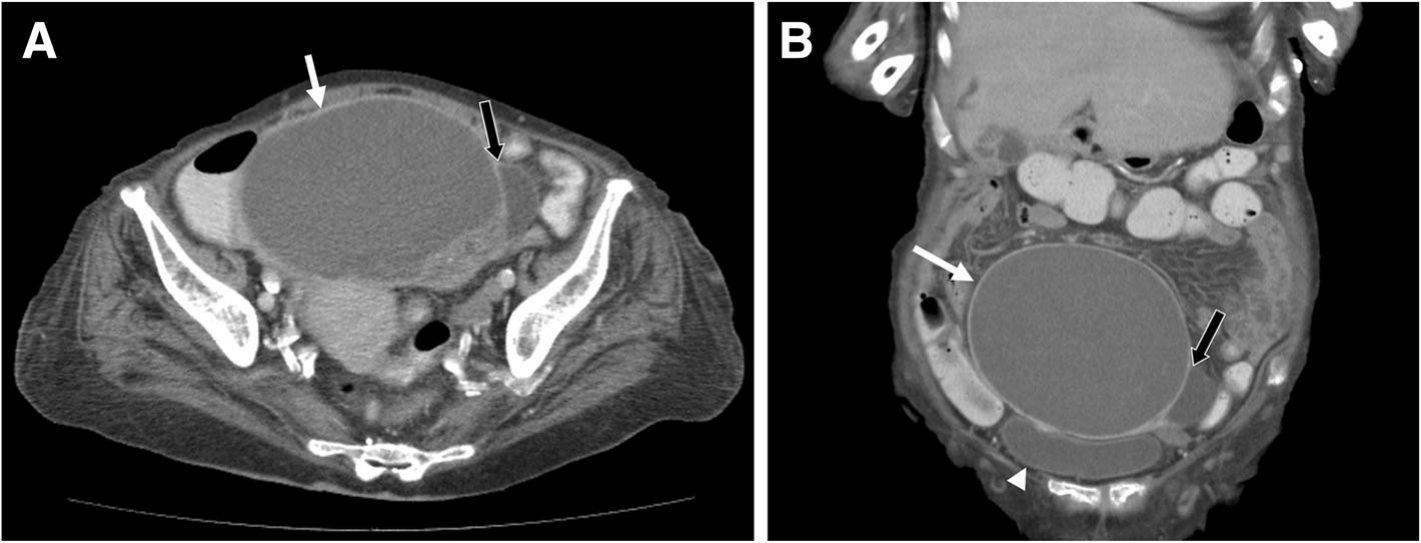
An abdominal computed tomography demonstrated the presence of a huge cystic mass 13.5 × 11.8 cm (white arrows) with internal septation (panel **a**, black arrows) and solid mural component. The urinary bladder (panel **b**, arrowhead) was compressed by it. No significant enlarged lymph nodes were found

After two weeks of antibiotic treatment, leukocytosis, pyuria, and sepsis resolved, but intermittent fever lasted along with pelvic tenderness. We thus consulted the gynecologist again, who then arranged an urgent abdominal computed tomography (CT) because of the highly possible surgical requirement upon consultation. The CT scan disclosed the presence of a huge cystic mass 13.5 × 11.8 cm with internal septation and mural solid component without any obvious fat stranding at lower abdomen nor any evidence of acute colitis. The urinary bladder was compressed by it (Fig. 1). No significant enlarged lymph nodes were found. Mucinous cystadenoma with ovarian torsion was suspected, and thus surgical intervention was arranged. During the surgery, a 12 × 10 × 10 cm right tubo-ovarian abscess with 800 mL of pus-like content was drained. Right salpingo-oophorectomy and pus culture were performed. The pathological examination showed ovarian tissue with acute and chronic inflammation, inflammatory exudate, and granulation tissue formation, which were compatible with that of a tubo-ovarian abscess, and its pus culture yielded *Escherichia coli*. Antibiotics were administered based on the culture sensitivity test, and her infection ultimately resolved thereafter.

## Discussion and conclusions

In a case series which enrolled 80 tubo-ovarian abscess patients, their age ranged from 15 to 69 years old with an average of 42 years old [1]. In another retrospective study enrolled 63 patients with a surgically confirmed tubo-ovarian abscess, only nine patients were post-menopausal [2], contrasting the rarity of our case who is a nonagenarian. Similarly, literature regarding tubo-ovarian abscess showed that the average age ranges 52–58-year-old in the post-menopausal group, as shown in Table 1 [1–10]. The risk factors for tubo-ovarian abscess include age between 15 to 25 years old, a prior history of pelvic inflammatory disease, and multiple sexual partners.

**Table 1:** Literature review of tubo-ovarian abscess in post-menopausal women

No.	Patient number (Post-menopausal/Total)	Age (year)	Microorganism	Treatment	Treatment outcome	Conclusion	Ref.
1	1/1	91	*Escherichia coli*	Exploratory laparotomy with antibiotics	Successful and patient survived	TOA could occur in nonagenarian women, especially those who are immunocompromised, which requires timely management for a better prognosis	Our case
2	1/1	55	*Clostridium perfringens*	Exploratory laparotomy with hysterectomy	Successful and patient survived	*Clostridium perfringens* can cause adnexal infection in the absence of trauma	8
3	1/1	71	N/A	Exploratory laparotomy	Successful and patient survived	Chronic TOA may rupture or fistulize to adjacent organs into the ischiorectal space	7
4	9/63	Pre-menopausal: 26 Post-menopausal: 52	N/A	Exploratory laparotomy	Successful and patient survived	An attempt at early recognition and surgical management of TOA is vital in post-menopausal women	2
5	17/80	Overall: 42	Anaerobes; negative results	Exploratory laparotomy	Successful and patient survived	Fewer patients were hospitalized in Oslo for PID during the period of 2000–2002 compared with ten years earlier, but a higher percentage of patients had developed TOA compared with the first period (43% compared with 26%), indicating a changing clinical panorama of PID	1
6	17/93	Pre-menopausal: 34 Post-menopausal: 58	N/A	Exploratory laparotomy	Successful and patient survived	For post-menopausal women with TOAs, pelvic malignancy should be excluded. Conservative treatment has no place during the menopause	9
7	20/20	N/A	N/A	Total hysterectomy	Successful and patient survived	Early detection and treatment of unruptured TOA had less surgery-related complications and had a shorter mean length of hospitalization	10
8	25/296	Overall: 34.5 ± 10.3	N/A	Exploratory laparotomy; laparoscopic treatment; broad-spectrum antibiotics	Successful and patient survived	Post-menopausal status on admission were associated with a failed response to conservative treatment	6
9	29/64	Early laparoscopic: 39.0 Conventional: 38.9	*Escherichia coli* *Peptostreptococci baumanmii*	Early laparoscopic treatment; conventional antibiotics	Successful and patient survived	Early laparoscopic treatment is associated with a shorter time of fever resolution, shorter hospitalization, and less blood loss compared with conventional treatment for TOA or pelvic abscess	4
10	35/318	Medical treatment: 35.6 ± 8.1 Medical + Surgical treatment: 37.3 ± 6.2	N/A	Exploratory laparotomy with drainage tube; conventional antibiotics	Successful and patient survived	The TOA size, complex multi-cystic mass image, CRP, and ESR are useful indicators as to whether surgical treatment is required for the management of TOA	5
11	39/144	Pre-menopausal: 38.5 ± 7.7 Post-menopausal: 54.3 ± 8.1	Group C *Streptococcus*	Exploratory laparotomy with antibiotics; drainage for premenopausal women only	One post-menopausal woman of TOA had malignancy, but no other women were diagnosed with cancer during a mean follow-up of 7.6 years	In post-menopausal women with TOA, the prevalence of concurrent pelvic malignancy was 2.6%, which is higher than in the general population, but lower than that reported in the literature; 44% were conservatively managed without any apparent cases of misdiagnoses of cancer	3

*Data were presented as mean or mean ± SD. Abbreviations: *N/A* not available, *TOA* tubo-ovarian abscess

Heaton et al. reported 20 post-menopausal women with a tubo-ovarian abscess in a case series; only 20% of patients were febrile, 45% presenting with leukocytosis, and 55% having a palpable pelvic mass [11]. In our patient, fever and leukocytosis were presented initially. However, due to her underlying dementia, the patient could not express her discomfort. Meanwhile, the initial physical examination did not reveal any acute abdominal sign, leading to delayed recognition of tubo-ovarian abscess in our case. Hsiao et al. analyzed 74 patients with surgically proved tubo-ovarian abscess, they found that an accurate preoperative diagnosis of the tubo-ovarian abscess was significantly lower in the post-menopausal group as compared to the pre-menopausal group (22% versus 54%), indicating a highly prevalent silent presentation of tubo-ovarian abscess in the post-menopausal group [12]. Also, another predisposing factor of our patient may be her immunocompromised status because of advanced age and uremia, usually manifesting as reduced antigen-presenting dendritic cells, depletion of naïve and central memory T cells and B cells, and impaired phagocytic function of neutrophils and monocytes [13].

Because rupture of a tubo-ovarian abscess is a life-threatening emergency, aggressive medical or surgical management is required immediately [14]. Therefore, during infection work-up, clinicians should always consider PID to avoid delayed management, even if patients are more than 70 years old, as is our patient. A complete infectious focus survey relies on a thorough physical examination as well as a pelvic examination. Also, the tubo-ovarian abscess should be listed as a differential diagnosis in all post-menopausal women, especially those who are immunocompromised or with a palpable pelvic mass, to enable timely management and better prognosis.

## Data Availability

All data presented in this report are included in this published article.

## Abbreviation

PID: Pelvic inflammatory disease
